# Non-alcoholic Fatty Liver Disease and Longitudinal Cognitive Changes in Middle-Aged and Elderly Adults

**DOI:** 10.3389/fmed.2021.738835

**Published:** 2022-01-17

**Authors:** Qi Liu, Chang Liu, Feifei Hu, Xuan Deng, Yumei Zhang

**Affiliations:** ^1^Department of Neurology, Beijing Tiantan Hospital, Capital Medical University, Beijing, China; ^2^Clinical Research Institute, Shanghai General Hospital, Shanghai Jiao Tong University School of Medicine, Shanghai, China; ^3^Department of Rehabilitation, Beijing Tiantan Hospital, Capital Medical University, Beijing, China; ^4^China National Clinical Research Center for Neurological Diseases, Beijing Tiantan Hospital, Capital Medical University, Beijing, China

**Keywords:** longitudinal study, non-alcoholic fatty liver disease, cognitive impairment, middle-aged and older populations, risk factors

## Abstract

**Background and Purpose:**

Non-alcoholic fatty liver disease (NAFLD) and cognitive impairment are common aging-related disorders. This study aims to explore the changes of cognitive function in middle-aged and elderly population with NAFLD from a Jidong impairment cohort.

**Methods:**

A total of 1,651 middle-aged and elderly participants (>40 years) without cognitive impairment were recruited into the current study in 2015 and were followed up until to 2019. Abdominal ultrasonography was used for diagnosis of NAFLD. Global cognitive function was assessed with the Mini-Mental State Examination (MMSE). Cognitive impairment was defined as a score <18 for illiterates, a score <21 for primary school graduates, and a score <25 for junior school graduates or above. Multivariable regression analysis was performed to evaluate the association between NAFLD and the four-year cognitive changes.

**Results:**

Out of 1,651 participants, 795 (48.2%) of them had NAFLD in 2015. Cognitive impairment occurred in 241 (14.6%) participants in 2019. Patients with NAFLD had higher 4-year incidence of cognitive impairment than non-NAFLD patients did (17.7 vs. 11.7%, *p* < 0.001). Multivariable linear regression analysis showed significant association of baseline NAFLD with lower MMSE score in 2019 (β = −0.36, *p* < 0.05). Multivariable logistic analysis found that the adjusted odds ratio (OR) with 95% confidence interval (CI) of baseline NAFLD was 1.45 (1.00–2.11) for cognitive impairment in 2019 (*p* = 0.05). We also identified effects of baseline NAFLD on subsequent cognitive impairment as modified by age (interaction *p* < 0.01) and carotid stenosis (interaction *p* = 0.05) but not by gender.

**Conclusions:**

NAFLD is associated with cognitive decline, especially in middle-aged and with carotid stenosis population.

## Introduction

Cognitive impairment is a leading cause of disability worldwide and imposes a heavy public and economic burden (1). The prevalence of cognitive impairment ranges from 9.7 to 23.3% among subjects aged 65 years or older in China (2). It is necessary to identify and to control its risk factors. Non-alcoholic fatty liver disease (NAFLD) is the most common chronic liver disease with a prevalence of 20–42% in China (3, 4). Previous studies demonstrated that NAFLD is not only confined to the liver but also harmful to the extra-hepatic multisystem (5, 6). Patients with NAFLD had reduced total cerebral brain volume, higher white mater hyperintensities, and more lacunar infarction than those without NAFLD (7–10).

A population with NAFLD were more likely to have impaired cognitive performance as compared with a healthy population (11–14). However, most of the previous studies were cross-sectional, which cannot assess the long-term effect of NAFLD on cognitive function (15, 16). In addition, it was also unexplored whether the effect of NAFLD on subsequent cognitive impairment is modified by age, gender and carotid stenosis. Therefore, we hypothesized that NAFLD had an independent detrimental effect on subsequent cognitive function. In current study, our aim was to evaluate the association between NAFLD and the longitudinal cognitive changes from 2015 to 2019 in middle-aged and elderly population from a Jidong cognitive impairment cohort.

## Methods

### Study Participants

Participants were derived from a Jidong cognitive impairment cohort study in 2015 and were followed for 4 years until 2019. A detailed information about this cohort were found in previous publication (17). Briefly, this cohort was established in April 2012 and the follow-up will continue until December 2024. This community-based study aims to investigate the incidence of cognitive impairment and its prognostic factors. Participants who met the following criteria were enrolled into the study: age 40 years or older, no history of dementia, and signed informed consent as provided. In accordance with the declaration of Helsinki, the study was approved by the Ethics Committee of Kailuan General Hospital of Tangshan City and the Medical Ethics Committee, Staff Hospital, Jidong Oilfield Branch, China National Petroleum Corporation.

### Data Collection

Baseline information including demographic characteristics, medical history, and biochemical variables were collected by a series of face-to-face standardized questionnaires, clinical examinations, and laboratory tests. Medical history included metabolic syndrome, hypertension, diabetes, hyperlipidemia, cardiovascular adverse events, and carotid stenosis. Hypertension was defined as self-reported history, any current use of antihypertensive drug, or a diagnosis of hypertension in a healthcare examination. Diabetes was defined as fasting glucose level ≥7.0 mmol/L, any current use of glucose-lowering drugs, or a self-reported history. Hyperlipidemia was defined as serum levels of triglyceride ≥1.7 mmol/L, total cholesterol ≥5.72 mmol/L, high-density lipoprotein ≤ 0.9 mmol/L, current use of lipid-lowering therapy, or a self-reported history. Cardiovascular adverse event included stroke, coronary heart disease, and heart failure. Metabolic syndrome was assessed using standardized definition (18). Carotid stenosis was defined with ultrasonography, that the internal carotid artery peak systolic velocity was >125 cm/s (19). Height, weight, and waist circumference of the participants were measured while in a relaxed standing. Body mass index (BMI) was calculated as weight in kilograms divided by height in meters squared.

### NAFLD Diagnosis

The diagnosis of NAFLD in this study was based on the guideline proposed by the Asia-Pacific Working Party (20). According to criteria, NAFLD was diagnosed on the presence of at least 2 of the following characteristics in the absence of other liver diseases or excessive alcohol: diffusely increased echogenicity of liver relative to kidney or spleen, hepatic vascular blurring and deep attenuation signs. Excessive alcohol consumption in this study was defined as >20 g/day for men and 10 g/day for women. Liver ultrasound examination was performed by using a B-mode Doppler sonography machine with a 3.5 MHz probe (ACUSON X300, Siemens, Germany). Imaging interpretation was accomplished by two expert radiologists who were unaware of the patients' health data.

### Outcome Measure

Follow-up was done by a face-to-face interview. All participants experienced the Chinese version of Mini-Mental State Examination (MMSE), so as to assess global cognitive status and to evaluate cognitive impairment incidence. MMSE, one of the most common and popular global cognitive examination, is used to evaluate five cognitive domains: orientation, registration, attention and calculation, recall, and language (21). The MMSE scores ranged from 0 to 30 points, and the higher scores indicated better cognitive functioning. Cognitive impairment was defined as the education-based cutoffs of MMSE score: <18 for illiterates, <21 for primary school graduates, and <25 for junior school graduates or above (21).

### Statistical Analysis

Continuous variables were presented as means ± standard deviations and categorical variables as frequency (percentage). The baseline characteristics of NAFLD group and healthy group were compared using independent sample *t*-tests, while the Mann-Whitney U tests were used for continuous variables, and the Chi-squared tests for categorical variables. The associations of NAFLD, with MMSE score and cognitive impairment, were assessed *via* multivariable linear and logistic regression analysis, respectively. In the first model, we just adjusted the sex, age, and educational levels. In the second model, we further adjusted the following covariates which have been demonstrated to be associated with cognitive function: BMI, hypertension, diabetes mellitus, hyperlipidemia, and carotid artery disease. The interaction of NAFLD with age, sex and carotid stenosis were also analyzed using a multivariate logistic model. *p* < 0.05 was considered to be statistically significant (2-sided). All analyses were performed with SAS version 9.4 (SAS Institute, Cary, North Carolina, USA).

## Results

### Study Participants and Characteristics

**Figure 1:**
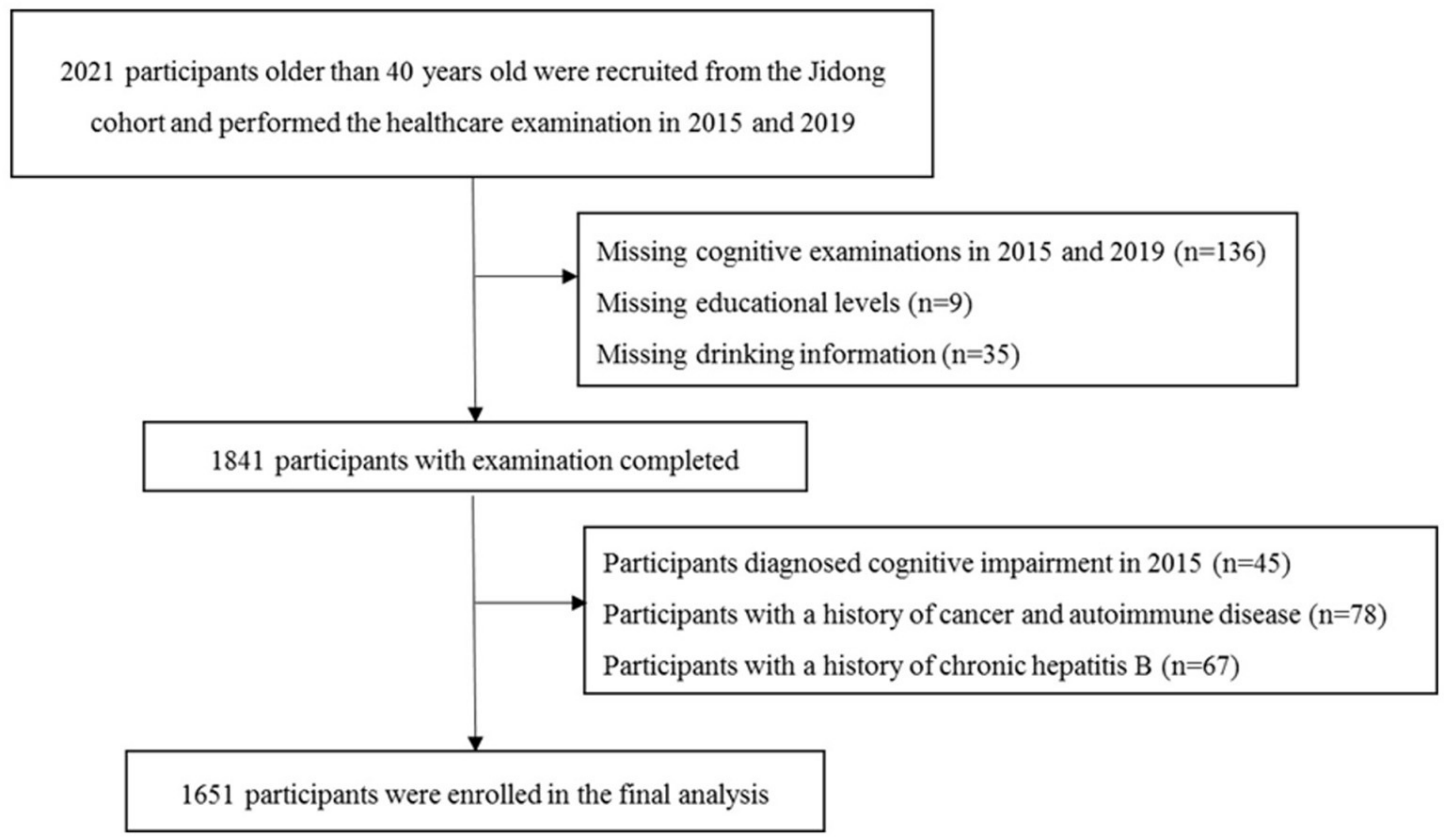
Flow chart of this study.

In total, there were 2,021 participants recruited into the Jidong study and had their healthcare examination in 2015 and 2019. We excluded 180 participants (8.9%) due to missing data, such as educational level, drinking habits, and cognitive performance; 145 (7.2%) due to a history of tumor, autoimmune disease, and chronic hepatitis B; and 45 (2.2%) due to a history of cognitive impairment. Finally, 1,651 participants were included into the current study (Figure 1). The baseline demographic characteristics and clinical features of the participants included and excluded in the final analysis were not significantly different, except the participants who were included were younger and had a higher educational level (Supplementary Table 1).

Non-alcoholic fatty liver disease (NAFLD) occurred in 795 participants (48.2%) in 2015 alone. Baseline characteristics are summarized in Table 1. Participants with NAFLD were older, more likely to be male, have higher BMI, higher levels of liver enzymes, blood glucose, serum lipid, higher proportion of history of diabetes mellitus, hypertension, hyperlipidemia, and carotid artery disease, as compared with those without NAFLD. Educational level and history of cardiovascular-adverse events were not significantly different between participants with or without NAFLD.

**Table 1:** Clinical characteristics of participants with or without NAFLD.

**Characteristics**	**Overall**	**NAFLD**	**No NAFLD**	***P* value**
	**(*n* = 1651)**	**(*n* = 795)**	**(*n* = 856)**	
Age (years)	53.4 ± 8.4	54.4 ± 8.2	52.4 ± 8.5	< 0.0001
Male (*n*, %)	806 (48.8)	434 (54.6)	372 (43.5)	< 0.0001
Educational level (years)				0.69
Elementary or below	95 (5.8)	48 (6.0)	47 (5.5)	
Middle school	103 (6.2)	53 (6.7)	50 (5.8)	
High school or above	1453 (88.0)	694 (87.3)	759 (88.7)	
BMI (kg/m^2^)	25.1 ± 5.6	26.6 ± 3.0	23.6 ± 6.9	< 0.0001
Waist (cm)	88.2 ± 22.1	92.1 ± 8.5	84.5 ± 29.2	< 0.0001
Smoking (*n*, %)	389 (23.6)	196 (24.7)	193 (22.6)	0.31
Metabolic syndrome (*n*, %)	609 (36.9)	430 (54.1)	179 (20.9)	< 0.0001
Hypertension (*n*, %)	592 (35.9)	368 (46.3)	224 (26.2)	< 0.0001
Diabetes (*n*, %)	209 (12.7)	150 (18.9)	59 (6.9)	< 0.0001
Hyperlipidemia (*n*, %)	940 (56.9)	570 (71.7)	370 (43.2)	< 0.0001
Cardiovascular adverse events (*n*, %)	28 (1.7)	17 (2.1)	11 (1.3)	0.18
Carotid stenosis (*n*, %)	221 (18.5)	127 (21.7)	94 (15.4)	< 0.005
Fast glucose (mg/dl)	111.7 ± 25.2	116.9 ± 30.2	106.9 ± 17.9	< 0.0001
Total cholesterol (mmol/L)	5.2 ± 1.0	5.3 ± 1.0	5.1 ± 0.9	< 0.0001
TG (mmol/L)	2.0 ± 1.5	2.5 ± 1.7	1.6 ± 1.2	< 0.0001
HDL-C (mmol/L)	1.3 ± 0.3	1.2 ± 0.3	1.3 ± 0.3	< 0.0001
LDL-C (mmol/L)	3.4 ± 0.8	3.5 ± 0.8	3.3 ± 0.8	< 0.0001
ALT (U/L)	20.5 ± 12.6	23.3 ± 13.9	17.8 ± 10.6	< 0.0001
AST (U/L)	21.8 ± 8.1	22.5 ± 8.2	21.1 ± 7.8	< 0.001
GT (U/L)	26.0 ± 24.6	29.4 ± 26.6	22.7 ± 22.0	< 0.0001
ALP (U/L)	94.6 ± 32.8	97.9 ± 26.7	91.5 ± 37.5	< 0.0001
Total bilirubin (mg/dl)	16.5 ± 7.2	16.4 ± 7.1	16.6 ± 7.3	0.61
Creatinine (μmmol/L)	97.2 ± 20.5	97.8 ± 18.6	96.6 ± 22.1	0.22

*NAFLD, nonalcoholic fatty liver disease; BMI, body mass index; TG, triglycerides; LDL-C, low-density lipoprotein cholesterol; HDL-C, high-density lipoprotein-cholesterol; ALT, alanine aminotransferase; AST, aspartate transaminase; GT, glutamine transferase; ALP, alkaline phosphatase*.

### Cognitive Function Measures in 2015 and in 2019 for Patients With NAFLD

Table 2 shows the cognitive performance in 2015 and in 2019 of the participants with or without NAFLD. No significant difference was found between groups on cognitive performance in 2015 (28.4 ± 1.6 vs. 28.6 ± 1.5, *p* = 0.07). Participants with NAFLD had higher proportion of cognitive impairment in 2019 (26.7 ± 3.0 vs. 27.2 ± 2.8, *p* < 0.0001) and faster cognitive decline from 2015 to 2019 (1.9 ± 2.6 vs. 1.5 ± 2.4, *p* < 0.001) than those participants without NAFLD. Cognitive impairment occurred in 241 (14.6%) participants in 2019. Patients with NAFLD had higher 4-year incidence of cognitive impairment than non-NAFLD patients did (17.7 vs. 11.7%, *p* < 0.001) (Table 2).

**Table 2:** Cognitive function in 2015 and 2019 in participants with or without NAFLD.

**Characteristics**	**Overall**	**NAFLD**	**No NAFLD**	***P* value**
	**(*n* = 1651)**	**(*n* = 795)**	**(*n* = 856)**	
MMSE score in 2015	28.5 ± 1.5	28.4 ± 1.6	28.6 ± 1.5	0.07
MMSE score in 2019	26.9 ± 2.9	26.7 ± 3.0	27.2 ± 2.8	< 0.0001
Cognitive decline from 2015 to 2019	1.7 ± 2.5	1.9 ± 2.6	1.5 ± 2.4	< 0.001
Cognitive impairment incidence from 2015 to 2019 (*n*, %)	241 (14.6)	141 (17.7)	100 (11.7)	< 0.001

*NAFLD, nonalcoholic fatty liver disease; MMSE, Mini-Mental State Examination*.

### Association Between NAFLD and Cognitive Function

Unadjusted analysis showed baseline NAFLD was significantly associated with lower MMSE score in 2019 (β = 0.58, *p* < 0.0001) and cognitive function declined from 2015 to 2019 (β = 0.45, *p* < 0.001). Multivariable analysis has still demonstrated significant association of baseline NAFLD with lower MMSE score in 2019 (β = −0.36, *p* < 0.05). We also found the trend of NAFLD with cognitive function decline from 2015 to 2019 (β = 0.29, *p* = 0.08) (Table 3).

**Table 3:** Association between baseline NAFLD and MMSE score from 2015 to 2019 in participants.

	**β coefficients**	**Standard error**	**95% confidence interval**	***P* value**
**MMSE score in 2019**				
Unadjusted	−0.58	0.14	−0.85 to −0.31	< 0.0001
Model 1	−0.38	0.13	−0.63 to −0.13	< 0.01
Model 2	−0.36	0.18	−0.71 to −0.01	< 0.05
**Cognitive decline from 2015 to 2019**				
Unadjusted	0.45	0.12	0.21 to 0.68	< 0.001
Model 1	0.34	0.12	0.10 to 0.58	< 0.01
Model 2	0.29	0.17	−0.04 to 0.62	0.08

*Model 1 adjusting following variables, age, sex, educational levels; Model 2 adjusting for age, sex, educational levels, body mass index, hypertension, diabetes mellitus, hyperlipidemia, carotid stenosis. MMSE, Mini-Mental State Examination*.

The unadjusted odds ratios (ORs) with 95% confidence interval (CI) of NAFLD were 1.47 (1.10–1.96) for cognitive impairment in 2019 (*p* < 0.001). The adjusted ORs with 95% CI were 1.45 (1.00–2.11) for cognitive impairment (*p* = 0.05) (Table 4).

**Table 4:** Association between baseline NAFLD and cognitive impairment in 2019 among participants.

	**Cognitive impairment (** ***n*** **, %)**		**Odds ratio (95% confidence interval)**			***P* interaction value**
	**NAFLD**	**No NAFLD**	**Unadjusted**	**Adjusted**		
Overall	141 (17.7)	100 (11.7)	1.63 (1.24–2.15)	1.45 (1.00–2.11)	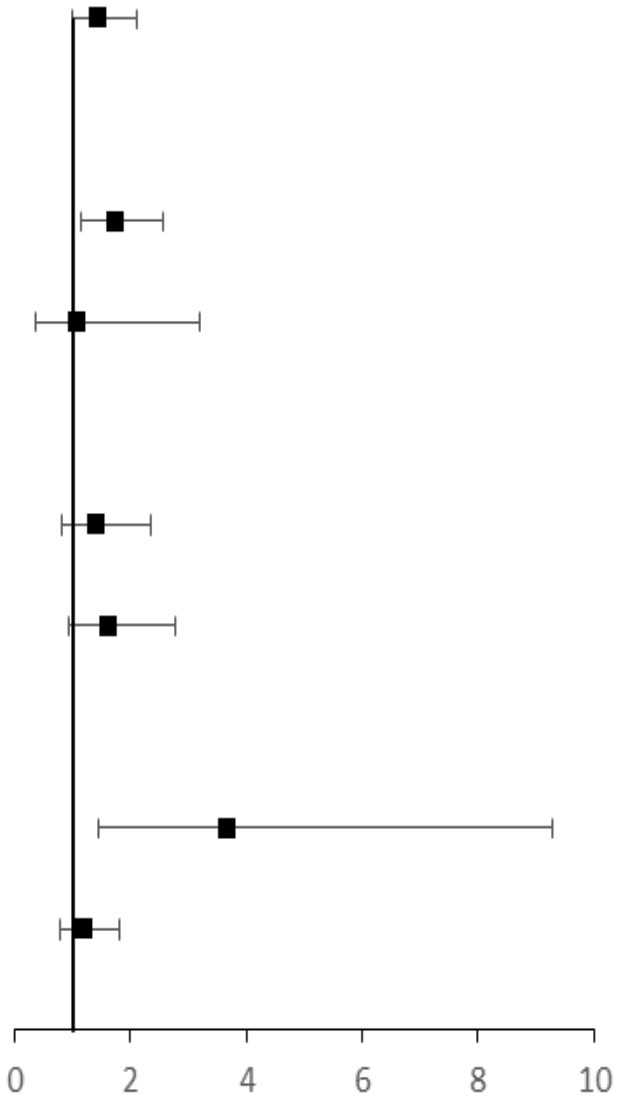	
Age (years)						< 0.01
40–65	119 (16.6)	84 (10.8)	1.66 (1.23–2.24)	1.72 (1.15–2.57)		
≥ 65	22 (17.5)	16 (21.3)	1.40 (0.67–2.93)	1.06 (0.35–3.20)		
Gender						< 0.05
male	75 (17.3)	50 (13.4)	1.35 (0.91–1.98)	1.39 (0.82–2.35)		
female	66 (18.3)	50 (10.3)	1.94 (1.31–2.89)	1.60 (0.93–2.76)		
Carotid stenosis and plaque						0.05
Yes	32 (25.2)	8 (8.5)	3.62 (1.58–8.29)	3.66 (1.44–9.29)		
No	75 (16.4)	67 (12.9)	1.32 (0.92–1.88)	1.19 (0.78–1.81)		

*Covariates included age, sex, carotid stenosis, educational levels, body mass index, hypertension, diabetes mellitus, and hyperlipidemia*.

### Subgroup Analysis According to Age, Gender, and Carotid Stenosis

In 2019, we assessed association of baseline NAFLD with cognitive in participants stratified by age, sex, and carotid stenosis (Table 4). The adjusted ORs with 95% CI were 1.72 (1.06–2.21) in middle-aged participants (40–65 years) and 1.06 (0.35–3.20) in elderly participants (≥ 65 years) (interaction *p* < 0.01). The adjusted ORs with 95% CI were 3.66 (1.44–9.29) in participants with carotid stenosis and 1.19 (0.78–1.81) in those without carotid stenosis (interaction *p* = 0.05). In addition, we did not identify statistically significant interaction between sex and NAFLD for cognitive impairment.

## Discussion

We found that a 4-year incidence of cognitive impairment was about 15.0% in our whole cohort study, but was ~18.0% in participants with NAFLD. Patients with NAFLD had 1.5-time increased risk of a subsequent 4-year cognitive impairment as compared to those without NAFLD. We also identified effects of baseline NAFLD on subsequent cognitive impairment as significantly modified by age and carotid stenosis but not by gender. To our knowledge, this is the first longitudinal study that investigated the relationship between NAFLD and cognitive function in a large community-dwelling of middle-aged and elderly population.

In the present study, NAFLD population had a higher 4-year incidence of cognitive impairment than the general population did. Also, the multivariable analysis of our study illustrated that NAFLD was independently associated with subsequent cognitive function, which was consistent with previous studies (11–14). Sang and colleagues firstly identified that NAFLD was significantly associated with worse cognitive performance in a large population-based study which contained 4,254 subjects (12). In a 3-year follow-up study, Elliott and colleagues demonstrated that patients with NAFLD had faster cognitive decline compared to the general population (11). However, other studies have failed to demonstrate such a relationship (15, 16). It remains open to question whether the relationship is an epiphenomenon. As liver is a regulator of systemic metabolic homeostasis and plays a critical role in the metabolism of glucose and lipid, greater risk for metabolic diseases (such as hypertension, diabetes, and hyperlipidemia) was found among patients with NAFLD in our study, which are related to both NAFLD and cognition (14, 22).

In the general population, few studies demonstrated that NAFLD is a risk factor for cognitive impairment (13, 23). In the multivariable analysis of our study, we found patients with NAFLD had a 1.5 folded-up risk of the subsequent 4-year cognitive impairment as compared to those without NAFLD. The mechanisms underlying the association of NAFLD with cognition are hard to elucidate. Insulin resistance was speculated as one of the possible mechanisms (24, 25). Pre-clinical experiment in the murine NAFLD model showed that insulin resistance has led the impaired activity of neurotransmitters enzymes and has limited the energy production accompanied by oxidative and endoplasmic reticulum stress, thereby, triggering a cascade of neurodegenerative changes (26–29). In addition, previous study found that patients with NAFLD had lower scavenging efficiency of central Amyloid-beta (Aβ), which is implicated with synaptic loss and neuronal degeneration (30). The low-density lipoprotein receptor-related protein-1 (LRP1) is one of the transporters involved in the clearance of Aβ out of the brain. Reduced LRP1 expression in patients with NAFLD leads to decreased peripheral Aβ protein clearance, which ultimately exacerbates amyloid load (25, 30). Moreover, liver can also regulate neuroendocrine system by synthesizing and secreting hepatic factors (31). For example, Irisin can accelerate neuroregeneration by promoting the production of neurotrophic factors (32), while the fetuin-A is a neuroprotective and anti-inflammatory molecule (33). The expression of both hepatokines decreases when fibrosis and fatty liver degeneration occurs (31).

Few studies have explored the effects of NAFLD on cognitive impairment as modified by age, gender, and carotid stenosis. In the present study, baseline NAFLD was associated with risk of a subsequent 4-year cognitive impairment in middle-aged but not in elderly population. A possible explanation is that the elderly was with more metabolic comorbidities than the middle-aged group, consequently, blunted the effect of NAFLD on cognition. We also identified the stronger effect of NAFLD on cognitive impairment in participants with carotid stenosis. Liver steatosis is characterized by a pro-inflammatory state, which promotes atherosclerosis and endothelial dysfunction, and, finally, induces micro- and macrovascular structural alterations, thereby, increasing the risk for vascular dementia (24, 34, 35). Previous studies confirmed that women had a higher incidence of dementia compared with men (36, 37). However, we did not find the interaction of gender and of NAFLD for cognitive impairment, which may be caused by the small sample size. It will be interesting in the future to observe the effects of gender, as well as other factors, on the association between NAFLD and cognitive impairment in a study with larger sample size.

Our study has several limitations. First, as age and educational level are associated with cognition (38), participants included in our analysis were generally younger and had a higher educational level compared with participants excluded, which may lead to an underestimation of cognitive impairment incidence. Second, we did not perform liver biopsy in this dwelling population. However, a meta-analysis found that ultrasound diagnosis of NAFLD had 84.8% sensitivity and 93.6% specificity compared with histology, suggesting that ultrasound is a reliable method for NAFLD diagnosis (39). In addition, because this was a *post hoc* analysis of an existing database, we were limited by the available data and were not able to explore further the effect of the severity of liver function and of liver fibrosis on cognitive decline. Finally, MMSE, which was used to assess cognitive status in this study, is a simple screening test and has a ceiling effect to identify cognitive decline (40). To detect cognitive dysfunctions more sensitively and to evaluate the characteristic of impaired cognitive domains in NAFLD population, other systematic cognitive tests, such as the Montreal Cognitive Assessment scale (MoCA), should be performed in the future research.

Non-alcoholic fatty liver disease (NAFLD) was associated with subsequent cognitive function, especially in middle-aged and with carotid stenosis population. Our findings suggested that NAFLD, which can be improved through lifestyle changes, can be a target for prevention of cognitive impairment.

## Data Availability Statement

The original contributions presented in the study are included in the article/Supplementary Material, further inquiries can be directed to the corresponding author/s.

## Ethics Statement

The studies involving human participants were reviewed and approved by the Ethics Committee of Kailuan General Hospital of Tangshan City and the Medical Ethics Committee, Staff Hospital, Jidong Oilfield Branch, China National Petroleum Corporation. The patients/participants provided their written informed consent to participate in this study.

## Author Contributions

YZ had full access to all of the data in the study, takes responsibility for the integrity of the data, and the accuracy of the data analysis. QL contributed to the study concept and drafted the paper. CL and YZ revised the manuscript for important intellectual content. FH and XD performed statistical analysis. All authors contributed to the article and approved the submitted version.

## Funding

Funding for this study was provided by the National Key R&D Program of China (2018YFC2002300, 2018YFC2002302, and 2020YFC2004102) and the National Natural Science Foundation of China (81972144, 31872785, and 81972148).

## Conflict of Interest

The authors declare that the research was conducted in the absence of any commercial or financial relationships that could be construed as a potential conflict of interest.

## Publisher's Note

All claims expressed in this article are solely those of the authors and do not necessarily represent those of their affiliated organizations, or those of the publisher, the editors and the reviewers. Any product that may be evaluated in this article, or claim that may be made by its manufacturer, is not guaranteed or endorsed by the publisher.
