# Supplementary material for: Non-alcoholic Fatty Liver Disease and Longitudinal Cognitive Changes in Middle-Aged and Elderly Adults
Source: Front Med (Lausanne). 2022 Jan 17;8:738835. doi: 10.3389/fmed.2021.738835 (PMC8803120; doi:10.3389/fmed.2021.738835)
Supplement: Supplementary file 1 [file Table_1.DOCX]

Supplementary Material

# Supplementary 1. Baseline characteristics between participants included and those excluded

| Characteristics | Participants included  (n=1651) | Participants excluded  (n=370) | | | P value |
| --- | --- | --- | --- | --- | --- |
| Age (years) | 53.4 ± 8.4 | | 55.7 ± 8.7 | <0.0001 | |
| Male (n, %) | 806 (48.8) | | 167 (45.1) | 0.20 | |
| Educational Level (years) |  | |  | <0.001 | |
| Elementary or below | 95 (5.8) | | 32 (8.7) |  | |
| Middle school | 103 (6.2) | | 42 (11.4) |  | |
| High school or above | 1453 (88.0) | | 296 (80.0) |  | |
| BMI (kg/m2) | 25.1 ± 5.6 | | 24.8 ± 3.4 | 0.43 | |
| Smoking (n, %) | 389 (23.6) | | 82 (22.2) | 0.57 | |
| Metabolic syndrome (n, %) | 609 (36.9) | | 146 (39.5) | 0.36 | |
| Hypertension (n, %) | 592 (35.9) | | 136 (36.8) | 0.74 | |
| Diabetes (n, %) | 209 (12.7) | | 60 (16.2) | 0.07 | |
| Hyperlipidemia (n, %) | 940 (56.9) | | 204 (55.1) | 0.53 | |
| Carotid stenosis and plaque (n, %) | 221 (18.5) | | 56 (20.6) | 0.42 | |
